# Non-Invasive Physical Plasma Regulates COX-2 via EP2/EP4 Expression in Periodontal Ligament Cells Under Inflammatory Conditions

**DOI:** 10.3390/ijms27156709

**Published:** 2026-07-27

**Authors:** Benedikt Eggers, Rami Kharroubi, Jana Marciniak, Svenja Beisel-Memmert, James Deschner, Franz-Josef Kramer, Matthias Bernhard Stope, Marjan Nokhbehsaim

**Affiliations:** 1 Department of Oral, Maxillofacial and Plastic Surgery, University Hospital Bonn, 53111 Bonn, Germany; franz-josef.kramer@ukbonn.de; 2 Section of Experimental Dento-Maxillo-Facial Medicine, Center of Dento-Maxillo-Facial Medicine, University Hospital Bonn, Welschnonnenstrasse 17, 53111 Bonn, Germany; rami4kharroubi@hotmail.de (R.K.); jana.marciniak@ukbonn.de (J.M.); m.saim@uni-bonn.de (M.N.); 3 Department of Orthodontics, University Hospital Bonn, 53111 Bonn, Germany; 4 Goethe University Frankfurt, Carolinum, Department of Orthodontics, 60596 Frankfurt am Main, Germany; beisel-memmert@med.uni-frankfurt.de; 5 Department of Periodontology and Operative Dentistry, University Medical Center of the Johannes Gutenberg University, 55131 Mainz, Germany; james.deschner@uni-mainz.de; 6 Department of Gynecology and Gynecological Oncology, University Hospital Bonn, 53111 Bonn, Germany; matthias.stope@ukbonn.de

**Keywords:** periodontitis, non-invasive physical plasma (NIPP), IL-1β, COX-2, PGE2 receptors, hPDLC

## Abstract

Periodontitis is a chronic inflammatory disease characterised by progressive destruction of the periodontal tissues and alveolar bone. Cyclooxygenase-2 (COX-2) plays a key role in the pathogenesis of inflammatory responses via prostaglandin E_2_ (PGE_2_) receptors (EP1–4). Non-invasive physical plasma (NIPP) has been shown to modulate cellular activity and exert antimicrobial and anti-inflammatory effects. The aim of this in vitro study was to investigate the impact of NIPP on inflammation- and apoptosis-related molecules, including COX-2, EP2-EP4, Interleukin (IL)-6, IL-8, apoptotic protease activating factor 1 (APAF-1), Caspase (CASP)-3, and CASP-9, IL-10 and B-cell lymphoma 2 (BCL2) in human periodontal ligament cells (hPDLC) under normal and inflammatory conditions. After exposure of hPDLC to IL-1β to mimic inflammation in vitro, cells were treated with NIPP. Gene expression was analysed 24 h post-treatment by quantitative RT-PCR. Protein levels were assessed by ELISA. NIPP significantly inhibited IL-1β-induced upregulation of *COX-2*, *EP2* and *EP4* receptors, and *APAF-1* mRNA expression in hPDLCs after 24 h. At the protein level, IL-1β-induced expression of COX-2, IL-6 and IL-8 was effectively attenuated by NIPP treatment. In conclusion, our data suggest that NIPP may modulate inflammatory responses in hPDLC, indicating its potential as a promising adjunctive approach for the treatment of periodontitis.

## 1. Introduction

Periodontitis is one of the most prevalent chronic inflammatory diseases worldwide and, alongside dental caries, represents the most common oral disease [1,2]. It is a multifactorial, inflammatory condition of the tooth-supporting tissues, including the gingiva, periodontal ligament, cementum, and alveolar bone [3]. Clinically, periodontitis is characterised by periodontal attachment and bone loss, increased probing pocket depths, and bleeding or pus on probing [4]. Smoking and diabetes mellitus are among the most influential risk factors contributing to disease severity [5].

At the cellular level, resident periodontal cells actively participate in the maintenance of tissue homeostasis and the orchestration of inflammatory responses. In this context, human periodontal ligament cells (hPDLC) play a pivotal role in preserving periodontal tissue integrity and mediating cellular responses to inflammatory stimuli [6,7]. As specialised connective tissue cells, they help to attach the tooth to the alveolar bone and regulate the production of a wide range of pro- and anti-inflammatory mediators [8,9]. However, under inflammatory conditions, the immunomodulatory capacity of PDL cells becomes dysregulated, thereby contributing to the amplification of periodontal inflammation and disease progression [7]. Consequently, hPDLC represent a key cellular population that can modulate the pathophysiology of periodontitis.

The inflammatory microenvironment characteristic of periodontitis is primarily initiated and sustained by dysbiotic subgingival biofilms, including *Porphyromonas gingivalis*, *Tannerella forsythia*, *Treponema denticola*, and/or *Aggregatibacter actinomycetemcomitans* [10,11,12,13]. These pathogens, their components such as Lipopolysaccharides, and/or products contribute a variety of substances, which trigger host immune responses in both soft and hard periodontal tissues. This process results in the release of pro-inflammatory mediators such as tumour necrosis factor-α (TNF-α), interleukin (IL)-1β, IL-6 and IL-8, as well as prostaglandin (PG)E_2_ and cyclooxygenase (COX)-2, mediated through activation of cell surface receptors, including prostaglandin E_2_ receptor (EP)1–EP4. These pathogen-induced inflammatory cascades are closely linked to the prostaglandin pathway, which plays a pivotal role in mediating periodontal tissue responses. In particular, prostaglandins are central to both physiological and pathophysiological processes in the periodontium [14]. They are synthesised by COX, also known as prostaglandin endoperoxide synthase [15]. Among the COX isoforms, COX-2 is a key inducible enzyme responsible for the conversion of arachidonic acid into prostaglandins and is strongly associated with inflammation, tissue destruction, pain, and disease progression in periodontitis [16]. Increased COX-2 expression has been reported in gingival tissues and gingival crevicular fluid at inflamed sites [16,17,18]. Collectively, these inflammatory processes contribute to osteoclast activation and subsequent alveolar bone resorption [16,19,20,21].

In addition to pro-inflammatory mediators, anti-inflammatory cytokines such as IL-10 also play a crucial role in regulating periodontal inflammation by suppressing excessive immune responses and thereby limiting tissue damage [22]. IL-10 has been demonstrated to suppress the production of pro-inflammatory cytokines and to modulate the activity of macrophages and T-cells, thus contributing to the resolution of inflammation [23].

Furthermore, the balance between cell survival and apoptosis is a critical determinant in periodontal tissue homeostasis. Anti-apoptotic proteins such as B-cell lymphoma 2 (BCL-2) are regulators of mitochondrial apoptosis pathways and contribute to cell survival under inflammatory conditions [24,25]. Dysregulation of apoptosis-related molecules, including reduced BCL-2 expression, has been associated with increased tissue destruction and disease progression in periodontitis [26]. Furthermore, cell survival is also regulated by pro-apoptotic molecules. Among these, apoptotic protease activating factor (APAF)-1 is a central adaptor protein that facilitates the formation of the apoptosome, leading to activation of initiator caspase (CASP)-9 and the executioner CASP-3, which together orchestrate programmed cell death. Altered expression of APAF-1, CASP-9, or CASP-3 has been linked to enhanced apoptosis in periodontitis [27].

Factors contributing to the onset and progression of periodontitis may likewise influence therapeutic outcomes. Accordingly, the successful management of periodontitis depends on multiple factors, including biofilm, host immune response, age, genetic predisposition, lifestyle, and systemic conditions [28]. Current periodontal therapy follows a structured, stepwise approach focusing on oral hygiene instruction, mechanical biofilm removal, surgical intervention when necessary, and long-term supportive periodontal care [29,30]. Despite these measures, controlling inflammation and preventing tissue destruction remain major clinical challenges, highlighting the need for adjunctive therapeutic strategies.

In recent years, non-invasive physical plasma (NIPP) has emerged as a promising adjuvant therapeutic approach for the management of chronic wounds, where it supports tissue repair and regeneration [31,32,33]. Its therapeutic applicability is attributed to its pronounced antimicrobial activity, its lack of cytotoxicity towards healthy tissue, and its ability to promote cell proliferation and viability [34,35,36,37]. Interestingly, NIPP exerts opposing effects on malignant cells [38,39,40]. Since the biological effects of NIPP strongly depend on the plasma source and application parameters, results obtained with different NIPP devices are not directly comparable [41,42]. Therefore, studies using clinically approved devices are essential to evaluate their specific biological effects and translational potential.

In dentistry, NIPP has demonstrated potent antimicrobial activity, the ability to reduce postoperative infections, and the potential to enhance wound healing in a painless, non-invasive manner [43,44,45,46]. While recent studies have reported beneficial effects of NIPP on regeneration-associated cellular functions of gingival fibroblasts and keratinocytes [36], its modulatory effects on pro-inflammatory actions of hPDLC remain poorly understood. Accordingly, this study aimed to assess the modulatory effects of NIPP on the expression of mediators associated with inflammation and apoptosis in hPDLC under inflammatory conditions.

## 2. Results

### 2.1. Spindle-Shaped Morphology and S100A4 Expression

The isolated cells from the periodontium demonstrated a spindle-shaped morphology and expressed S100A4, a marker for fibroblastic cells (Figure 1), indicating that hPDLC were obtained.

### 2.2. Effect of NIPP on Cell Metabolic Activity

Firstly, cell metabolic activity was assessed by an XTT assay following NIPP treatment in the presence and absence of IL-1β, which was used to mimic inflammatory conditions in vitro. Neither NIPP, IL-1β, nor their combination had a significant effect on metabolic activity in hPDLC (Figure 2). Nevertheless, stimulation of the cells with IL-1β caused a trend toward increased metabolic activity, which seemed to be counteracted after NIPP treatment.

### 2.3. In Vitro Wound Closure Assay

Secondly, we investigated whether NIPP modulates wound closure in the presence and absence of IL-1β, with wound closure serving as an indicator of the cells’ proliferative and migratory capacity. Notably, a stimulatory effect of NIPP on wound closure became evident 48 h after treatment. In contrast, IL-1β incubation markedly impaired wound closure in hPDLC, with the most pronounced reduction observed at 48 h. Importantly, simultaneous NIPP treatment partially counteracted the inhibitory effects of IL-1β, suggesting a restorative effect in inflammation-compromised cells (Figure 3 and Figure 4).

### 2.4. Effect of NIPP on Molecules Associated with Inflammation in hPDLC

Next, we examined the gene expression of *COX-2* and *EP2*–*EP4* in hPDLC. IL-1β upregulated the gene expression of *COX-2* as well as *EP2* and *EP4* (Figure 5a,b,d). Interestingly, this stimulatory effect of IL-1β was significantly suppressed by the simultaneous treatment with NIPP. NIPP alone had no significant effect on *COX-2*, *EP2*, or *EP4* expression. In contrast, however, treatment of the cells with NIPP alone led to a significant upregulation of *EP3*. Neither IL-1β nor the combination of IL-1β and NIPP had a significant influence on *EP3* gene expression (Figure 5c).

Furthermore, we also studied the impact of NIPP on other molecules related to inflammation, such as *TNF-α*, *IL-6* and *IL-8*. Following incubation with IL-1β, a significant upregulation of the mRNA expression of *TNF-α*, *IL-6* and *IL-8* was observed at one day (Figure 6a–c). As observed for *COX-2* and some of the EP receptors, the stimulatory effect of IL-1β was counteracted by simultaneous application of NIPP (Figure 6a–c). In addition, NIPP alone had an inhibitory effect on *IL-6* and *IL-8* expression.

### 2.5. Effect of NIPP on Molecules Associated with Inflammation on Protein Levels

Moreover, the effect of NIPP on inflammatory mediators was further evaluated at the protein level using ELISA. In line with the transcriptional data, protein analyses demonstrated that NIPP reduced COX-2, IL-6, and IL-8 protein levels in hPDLCs. ELISA measurements revealed a significant increase in COX-2 protein expression (Figure 7a) following incubation with IL-1β, which was counteracted by NIPP treatment. Comparable anti-inflammatory effects were observed for IL-6 and IL-8 (Figure 7b,c).

### 2.6. Effect of NIPP on Molecules Associated with Apoptosis in hPDLC

In addition, we investigated the effect of NIPP on apoptosis-associated molecules, namely *APAF-1*, *CASP-3*, and *CASP-9*. Stimulation with IL-1β resulted in a significant upregulation of the mRNA expression levels of *APAF-1*, *CASP-3*, and *CASP-9* after 24 h. This IL-1β-induced increase in mRNA expression was counteracted by similar treatment with NIPP. Notably, treatment with NIPP alone did not exert a significant effect on the mRNA expression of *APAF-1* and *CASP-9* after 24 h (Figure 8a–c).

### 2.7. Effect of NIPP on Anti-Inflammatory and Anti-Apoptotic Molecules in hPDLC

In contrast, two anti-inflammatory mediators were examined. Incubation with IL-1β resulted in a significant downregulation of *BCL-2* mRNA expression after 24 h (Figure 9b). This effect was counteracted by NIPP treatment. Notably, NIPP treatment alone significantly upregulated *IL-10* mRNA expression (Figure 9a).

## 3. Discussion

The present study demonstrates that NIPP modulates key inflammatory and apoptosis-related pathways in hPDLC under IL-1β-induced inflammatory conditions. Specifically, NIPP attenuated the expression of pro-inflammatory mediators at both transcriptional and protein levels, modulated the expression of apoptosis-related genes, and promoted cell migration in the scratch assay without compromising cell metabolic activity. Collectively, these findings demonstrate that NIPP modulates inflammatory responses, apoptosis-related gene expression, and cell migration in hPDLC under inflammatory conditions. These observations provide a rationale for further mechanistic investigations and subsequent preclinical studies evaluating the biological effects and therapeutic potential of NIPP in periodontal tissues.

Inflammation is a central driver of periodontal tissue destruction, and mediators such as COX-2, IL-6, IL-8, and TNF-α play key roles in amplifying the inflammatory response [14,16,19]. In the present study, IL-1β stimulation induced a marked upregulation of these mediators, confirming the validity of the in vitro inflammation model. Importantly, NIPP treatment significantly reduced IL-1β-induced expression of COX-2, IL-6, and IL-8 at both gene and protein levels, as well as *TNF-α* at the transcriptional level. The consistency observed between transcriptional and protein data strengthens the evidence for a robust anti-inflammatory effect of NIPP. These findings are in accordance with previous studies demonstrating that NIPP can modulate cytokine expression and inflammatory responses in oral cell types [47,48,49].

In addition to classical cytokines, the present study further demonstrates that NIPP influences the COX-2/PGE_2_ receptor axis. IL-1β-induced upregulation of *EP2* and *EP4* receptors was attenuated following NIPP treatment, whereas *EP3* expression was increased under non-inflammatory conditions. Given that EP2 and EP4 are generally associated with pro-inflammatory signalling [50], while EP3 is linked to inhibitory or anti-inflammatory pathways [51], these findings may indicate a shift towards a less pro-inflammatory receptor expression profile. Although the precise biological implications require further investigation, these findings suggest that NIPP suppresses inflammatory mediator production and may potentially influence downstream signalling pathways involved in periodontal inflammation.

Beyond its anti-inflammatory effects, NIPP also modulated the expression of apoptosis-related genes [49,52]. IL-1β stimulation increased the expression of APAF-1, CASP-9, and CASP-3, indicating activation of the intrinsic apoptotic pathway under inflammatory conditions. This effect was significantly attenuated following NIPP treatment. Concurrently, NIPP counteracted the IL-1β-induced downregulation of the anti-apoptotic molecule *BCL-2*. These findings indicate that NIPP modulates the expression of genes involved in apoptosis under inflammatory conditions. Taken together, these transcriptional changes suggest that NIPP may favour cell survival over apoptosis in periodontal cells exposed to an inflammatory environment. Such an effect could be of particular relevance in the context of periodontal tissue preservation, where excessive apoptosis contributes to tissue breakdown [53,54]. However, as no functional apoptosis assays were performed, these data should be interpreted as changes in apoptosis-related gene expression rather than direct evidence of altered apoptotic activity. Future studies employing functional apoptosis assays will be required to determine whether these transcriptional changes translate into altered apoptotic responses.

The observed upregulation of *IL-10* expression following NIPP treatment further supports an anti-inflammatory and regulatory role. IL-10 is a key cytokine involved in limiting excessive immune responses and promoting resolution of inflammation [23,55]. The simultaneous modulation of pro-inflammatory mediators, anti-inflammatory cytokines, and apoptosis-related molecules may indicate a coordinated immunomodulatory effect rather than targeting a single pathway.

In line with these molecular findings, functional assays demonstrated that NIPP enhanced cell migration, as reflected by improved wound closure. In contrast, IL-1β impaired wound closure, which is consistent with the known detrimental effects of inflammation on tissue regeneration [56,57]. Notably, NIPP partially counteracted this impairment. Importantly, NIPP did not adversely affect cell metabolic activity, indicating that the observed pro-migratory effects were not associated with reduced cell viability. Taken together, these findings suggest that NIPP may support regenerative cellular responses in an inflammatory microenvironment.

The observed anti-inflammatory and pro-migratory effects of NIPP may also be of interest in the context of adjunctive regenerative strategies currently used in periodontology [58]. Established approaches, such as enamel matrix derivative–based therapies, are used to support periodontal wound healing and tissue regeneration [59]. Although direct comparisons cannot be made based on the present in vitro findings, the multimodal effects observed following NIPP treatment may warrant further investigation in the context of regenerative periodontal therapies.

The biological effects of NIPP are likely mediated by reactive oxygen and nitrogen species, generated during treatment [60]. Previous characterisation of the plasma device used in the present study demonstrated the generation of hydrogen peroxide (H_2_O_2_), ozone (O_3_), nitrite (NO_2_^−^), and nitrate (NO_3_^−^) under comparable treatment conditions, while no biologically relevant temperature increase occurred during the applied treatment time [48]. These findings support the assumption that the observed biological responses are primarily mediated by plasma-derived reactive oxygen and nitrogen species rather than thermal effects. At controlled concentrations, RONS function as important signalling molecules that can modulate intracellular redox homeostasis and influence redox-sensitive signalling pathways involved in inflammation, cell survival, and migration [39,61]. In periodontal ligament cells, redox-sensitive signalling has been associated with the regulation of inflammatory responses, cytokine production, cell survival, and cell migration in response to oxidative stimuli. In particular, modulation of pathways such as NF-κB and MAPK may contribute to the reduced expression of pro-inflammatory mediators and the altered expression of apoptosis-related genes observed in the present study [62,63,64]. However, as intracellular RONS levels and downstream signalling pathways were not directly investigated, these mechanistic considerations remain hypothetical and require experimental validation in future studies.

From a methodological perspective, IL-1β was used as a well-established pro-inflammatory stimulus to model periodontal inflammation in vitro. Its ability to reproducibly induce key inflammatory pathways, including COX-2 and cytokine expression, makes it suitable for controlled experimental conditions [65,66]. Nevertheless, periodontal disease is characterised by complex host–pathogen interactions, and stimulation with bacterial components such as lipopolysaccharides or whole pathogens may provide additional insights [67,68,69]. Future studies should therefore include more complex inflammatory models, including combinations of microbial and host-derived stimuli.

The use of primary hPDLC derived from multiple donors represents a strength of the present study, as it increases the biological relevance compared with immortalised cell lines. Although inter-individual variability was observed, the overall response pattern remained consistent across the five donors. However, the limited number of donors may have reduced statistical power in some analyses. Increasing sample size in future studies may further strengthen the robustness of the findings.

Regarding NIPP application, the study employed an intraorally approved device allowing homogeneous exposure of cells under standardised conditions. While this approach ensures reproducibility, it does not fully reflect the spatially restricted application typically used in clinical settings [70,71]. Since the biological effects of NIPP are highly device-specific, results obtained with different plasma sources cannot be directly compared [41,42]. In addition, NIPP effects are known to depend on treatment parameters such as intensity, duration, and distance [71,72,73]. Therefore, the present findings should primarily be interpreted in the context of the specific device and treatment conditions used in this study. Systematic investigation of different NIPP devices and application settings will therefore be important for future translational studies.

Several limitations of the present study should be considered. First, the study was conducted in vitro and therefore cannot fully replicate the complex cellular and molecular interactions present in vivo. Consequently, the findings should be interpreted with caution regarding their direct clinical relevance. Second, although potential involvement of redox-sensitive signalling pathways such as NF-κB and MAPK was discussed, no direct mechanistic analyses were performed. Therefore, mechanistic interpretations remain speculative and should be validated in future studies using pathway-specific approaches. Third, only selected inflammatory mediators, including COX-2, IL-6, and IL-8, were assessed at both the transcriptional and protein levels, whereas the remaining targets were analysed exclusively at the mRNA level. Since changes in gene expression do not necessarily translate into corresponding alterations in protein abundance or biological activity, additional protein-based and functional analyses are required to validate these transcriptional findings. Fourth, only a single inflammatory stimulus (IL-1β), one concentration, and one time point were investigated. While IL-1β is a well-established model for inducing periodontal inflammation, periodontal disease is characterised by a more complex and dynamic inflammatory microenvironment [74]. Fifth, only a single cell type (hPDLC) was investigated. However, periodontal tissue homeostasis and disease progression involve interactions between multiple cell populations, including immune cells, epithelial cells, and bone-related cells, which were not represented in this model [75]. Sixth, although NIPP enhanced cell migration in the scratch assay, this experimental model primarily reflects collective cell migration and does not directly assess periodontal tissue regeneration. Furthermore, apoptosis-related conclusions are based on gene expression analyses only, as no functional apoptosis assays were performed. Therefore, further studies employing functional apoptosis assays as well as dedicated in vitro and in vivo regeneration models are required to confirm these findings. Finally, only a single set of NIPP treatment parameters was investigated. As the biological effects of NIPP are known to depend on treatment duration and application settings, future studies should include systematic dose–response experiments and evaluation of different treatment protocols to further optimise therapeutic application.

In conclusion, the present study demonstrates that NIPP modulates inflammatory responses in hPDLC under inflammatory conditions by reducing the expression of inflammatory mediators and altering the expression of apoptosis-related genes without impairing cell metabolic activity. Furthermore, NIPP enhanced cell migration in the scratch assay, suggesting a beneficial effect on the migratory behaviour of hPDLC under the experimental conditions applied. However, dedicated functional apoptosis assays and regeneration models are required to determine whether these transcriptional and cellular changes translate into anti-apoptotic or regenerative effects in vivo. The present findings provide a rationale for further mechanistic investigations and subsequent preclinical studies to better define the biological effects and therapeutic potential of NIPP in periodontal tissues.

## 4. Materials and Methods

### 4.1. Sample Collection

Primary hPDLC employed in this study were isolated from periodontally healthy teeth extracted for orthodontic reasons from five donors at the University Hospital of Bonn, Germany. The study was conducted in accordance with the Declaration of Helsinki and approved by the Ethics Committee of the University Hospital of Bonn (# 111/17). Written informed consent was obtained from all donors prior to sample collection. Extracted teeth were immediately transported in Dulbecco’s Modified Eagle Medium (DMEM; Invitrogen, Waltham, MA, USA) supplemented with 10% fetal bovine serum (FBS; Invitrogen) and 1% penicillin/streptomycin (Invitrogen). Following transportation, teeth were rinsed with 0.9% sodium chloride solution (Braun, Melsungen Germany). Subsequently, a small fragment of periodontal ligament tissue was carefully removed from the middle third of the root using a scalpel and placed in a sterile Petri dish (VWR, Radnor, PA, USA), overlaid with a coverslip. Two millilitres of growth medium were added, and the tissue was incubated at 37 °C in a humidified atmosphere containing 5% CO_2_ for 7–10 days. Outgrowing hPDLC were monitored and, upon sufficient proliferation, transferred to culture flasks (VWR) for trypsinization. The isolated hPDLC were characterised by immunofluorescence microscopy prior to their use in further experiments. For all experiments, hPDLC at passages 3–5 were used. Cells derived from the five individual donors were cultured, treated, and analysed separately. For all subsequent experiments, hPDLC from each donor were seeded separately at a density of 5 × 10^4^ cells per culture dish.

### 4.2. Staining of Cells

For fluorescence and cytochemical analyses, hPDLC were seeded onto glass coverslips (Thermo Fisher Scientific, Singapore) and cultured as described above. Upon reaching confluence, cells were fixed with 4% paraformaldehyde (Sigma-Aldrich, St. Louis, MO, USA) for 10 min, washed with phosphate-buffered saline (PBS), and permeabilised with 0.1% Triton™ X-100 (Sigma-Aldrich) for 5 min. For immunofluorescent characterisation, fixed cells were incubated overnight at 4 °C in a humidified chamber with a primary rabbit anti-S100A4 antibody (1:400; Abcam, Cambridge, UK) diluted in 1% bovine serum albumin (BSA). Subsequently, cells were rinsed with PBS and incubated with a goat anti-rabbit IgG (H&L) Alexa Fluor™ 488-conjugated secondary antibody (1:400; Abcam, Cambridge, UK) for 60 min at room temperature. Stained cells were acquired using the ZOE™ Fluorescent Cell Imager (Bio-Rad, Hercules, CA, USA) and the Axioskop 2 microscope (Carl Zeiss, Jena, Germany) with an AxioCam MRc camera and the AxioVision 4.7 software (Carl Zeiss), respectively.

### 4.3. Inflammatory Condition In Vitro

Recombinant human interleukin-1β (IL-1β; Calbiochem, San Diego, CA, USA), a key pro-inflammatory mediator associated with periodontitis, was employed to establish an in vitro model of cellular inflammation. Prior to NIPP stimulation, hPDLC were pre-incubated with IL-1β at a concentration of 1 ng/mL for up to 1 h, as described previously [66]. The subsequent 24 h incubation period used for gene expression and protein analyses was selected based on previous studies and preliminary experiments.

### 4.4. Non-Invasive Physical Plasma Application

To investigate the immunomodulatory effects of NIPP, hPDLC were first pre-stimulated with IL-1β. Subsequently, the cells were treated with NIPP using a plasma device (plasma MEDICAL SYSTEMS^®^ GmbH; Plasma One Dental-Set, Nievern, Germany) with a specific probe (PS30) for 120 s. The treatment duration was selected based on preliminary optimisation experiments comparing different exposure times (60, 120, and 180 s), with 120 s showing the most pronounced biological response under the experimental conditions used. The device was operated at an output voltage of 18 kV at the highest intensity setting (level 5) and has been comprehensively characterised previously [48], including determination of temperature development and plasma-generated reactive oxygen and nitrogen species under comparable experimental conditions. NIPP was applied directly to the cell cultures using ambient air as the working gas. Treatment was performed at a constant distance of 2 mm between the plasma source and the surface of the cell culture medium. Untreated cells served as the control group.

### 4.5. Gene Expression Analysis Real-Time PCR

To determine the levels of gene expression following the treatment of IL-1β-pre-stimulated human periodontal ligament cells with NIPP, total RNA was isolated using the RNeasy Mini Kit (Qiagen, Hilden, Germany) according to the manufacturer’s instructions. Complementary DNA (cDNA) was then synthesised using the iScript™ Select cDNA Synthesis Kit (Bio-Rad, Munich, Germany). Quantitative real-time PCR (qRT-PCR) was performed using reaction mixtures containing 1 µL of cDNA, 2.5 µL of commercially available, gene-specific primers for *APAF-1*, *BCL-2*, *CASP-3*, *CASP-9*, *COX-2*, *EP2*, *EP3*, *EP4*, *IL-6*, *IL-8*, *IL-10*, *TNF-α* and the housekeeping gene *GAPDH* (QuantiTect Primer Assay, Qiagen), 9 µL of deionised water and 12.5 µL of SsoAdvanced™ Universal SYBR^®^ Green Supermix (Bio-Rad). Amplification and detection were carried out using the iCycler iQ5 real-time PCR detection system (Bio-Rad). The thermal cycling conditions were as follows: an initial denaturation step at 95 °C for 5 min, followed by 40 cycles of denaturation at 95 °C for 10 s and a combined annealing/extension step at 60 °C for 30 s. Gene expression levels were analysed using the comparative threshold cycle method. RNA was isolated 24 h after NIPP treatment, as this time point was selected based on previous studies and preliminary experiments.

### 4.6. Protein Analysis

COX-2 protein levels were analysed in cell lysates, whereas IL-6 and IL-8 concentrations were determined in the corresponding cell culture supernatants using specific enzyme-linked immunosorbent assay (ELISA) kits (COX-2: DYC4198-2, Human/Mouse COX-2 DuoSet ELISA; IL-6: DY206, Human IL-6 DuoSet ELISA; IL-8/CXCL8: DY208, Human IL-8/CXCL8 DuoSet ELISA; R&D Systems, Abingdon, UK), according to the manufacturer’s instructions. Colourimetric detection was performed using a spectrophotometer (Epoch Microplate Spectrophotometer, BioTek Instruments, Winooski, VT, USA) at a wavelength of 450 nm. The obtained ELISA results were normalised to the total protein concentration determined using the Pierce™ BCA Protein Assay Kit (23227, Thermo Scientific, Pierce Biotechnology, Rockford, IL, USA), with absorbance measured at 570 nm. Protein analysis was performed 24 h after NIPP treatment, based on previous studies and preliminary experiments.

### 4.7. Cell Proliferation Assay (XTT Assay)

Cell metabolic activity was quantified using the XTT assay (Cell Proliferation Kit XTT, AppliChem, Darmstadt, Germany). IL-1β pre-stimulated hPDLC were treated with NIPP or left untreated and subsequently incubated for 24 h. A freshly prepared XTT reaction mixture was added to each experimental group in accordance with the manufacturer’s instructions. Specific absorbance was measured using a microplate reader (Epoch™ Microplate Spectrophotometer, BioTek Instruments) at an optical density of 450 nm with a reference wavelength of 690 nm. For quantification, background absorbance from culture medium without cells was subtracted, and the resulting values were expressed as cell metabolic activity (% of the untreated control).

### 4.8. Scratch Wound Assay

A scratch wound assay was performed to assess cell migration. Briefly, confluent monolayers of stimulated hPDLC were mechanically scratched using a sterile scraper, creating standardised 3 mm-wide defects as previously described [76]. Cells from each experimental group were then placed into an incubator equipped with JuLI™ Br Live Cell Analyzer (NanoEnTek, Seoul, Republic of Korea). Two Petri dishes per experimental condition with comparable wound widths and cell confluence were monitored in parallel for up to 96 h. Time-lapse images were automatically acquired every 30 min using the integrated camera of the JuLI Br microscope to monitor cell migration and subsequently quantified using JuLI Br PC software (version 1.0.0; NanoEnTek). Cell migration was calculated as the percentage of the initially cell-free wound area that had been repopulated by migrating cells. The observation period was selected based on previous studies and preliminary experiments to capture the complete kinetics of hPDLC migration.

### 4.9. Statistical Analysis

Statistical analyses were performed using GraphPad Prism version 10 (GraphPad Software, Inc., La Jolla, CA, USA). Measurements obtained from cells derived from the same donor under different experimental conditions were considered paired. Gene-expression and XTT data comprising four related experimental groups were analysed using the Friedman test followed by Dunn’s multiple-comparisons test. ELISA data comprising two paired experimental groups were analysed using the Wilcoxon matched-pairs signed-rank test. Scratch-assay data, including four experimental groups and repeated measurements over time, were analysed using repeated-measures two-way analysis of variance with Greenhouse–Geisser correction, followed by Tukey’s multiple-comparisons test. A *p*-value of <0.05 was considered statistically significant. Unless otherwise stated, all experiments were performed using hPDLC isolated from five independent donors. Cells derived from each donor were cultured and analysed separately throughout the study. For each experimental condition, independently seeded cell cultures were prepared and processed in parallel. The corresponding sample size (n) for each experiment is indicated in the respective figure legends and reflects the number of independently analysed cell cultures included in the statistical analysis. Differences in sample size between individual datasets resulted from occasional technical exclusions during data acquisition.

## 5. Conclusions

NIPP treatment positively modulates the inflammatory response in hPDLC by downregulating pro-inflammatory cytokines and apoptosis markers without compromising cell viability. Its effects involve the COX-2-associated EP2 and EP4 receptors, highlighting a regulatory mechanism for inflammation. Unlike in tumour cells, NIPP exerts protective, cell-supportive effects in hPDLC. These findings suggest that NIPP has potential as an adjunctive therapy in periodontitis, although further in vitro and in vivo studies are required to confirm its efficacy and long-term stability.

## Figures and Tables

**Figure 1:**
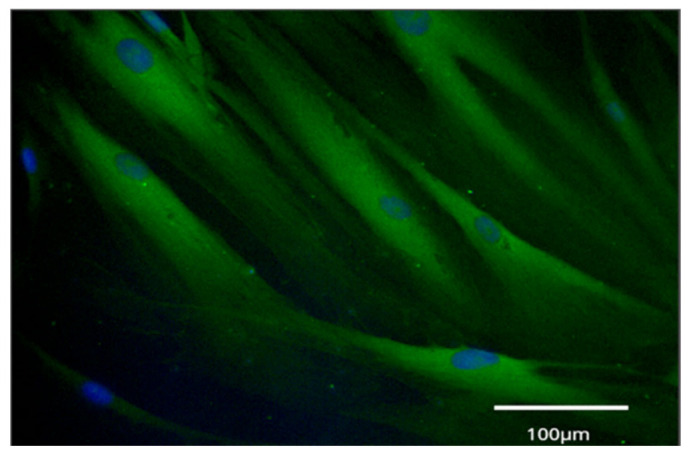
Immunofluorescent staining of S100A protein in human periodontal ligament (PDL) cells. S100A4 is shown in green, and the cell nuclei were counterstained with DAPI (blue). The scale bar represents 100 micrometres.

**Figure 2:**
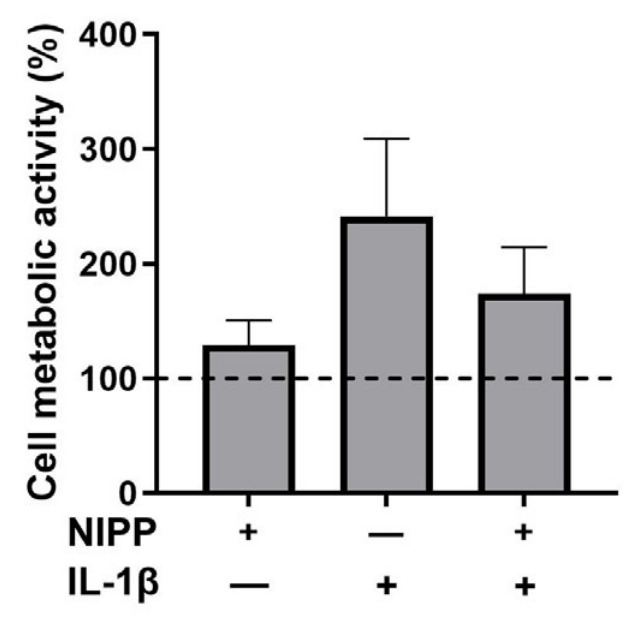
Cell metabolic activity (XTT) of hPDLC in the presence or absence of NIPP (120 s) and/or IL-1β (1 ng/mL) compared to the untreated control (dashed line) at 24 h. “+” indicates the presence and “−” the absence of the respective treatment. *n* = 15.

**Figure 3:**
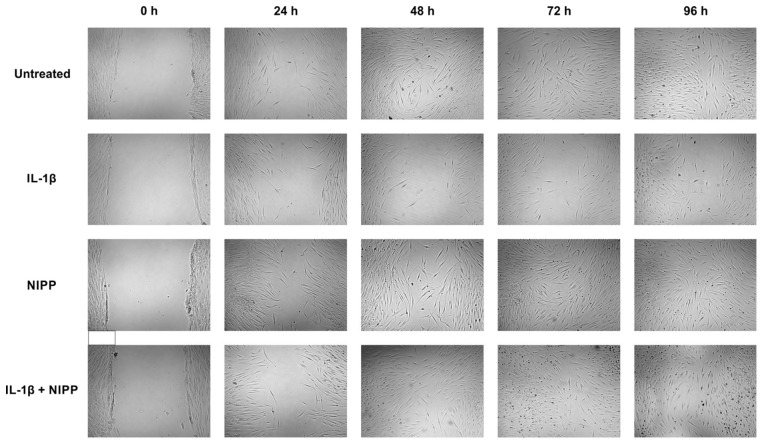
In vitro wound closure assay of hPDLC monolayers over 96 h. Representative images showing repopulation rate of hPDLC, assessed by scratch assay.

**Figure 4:**
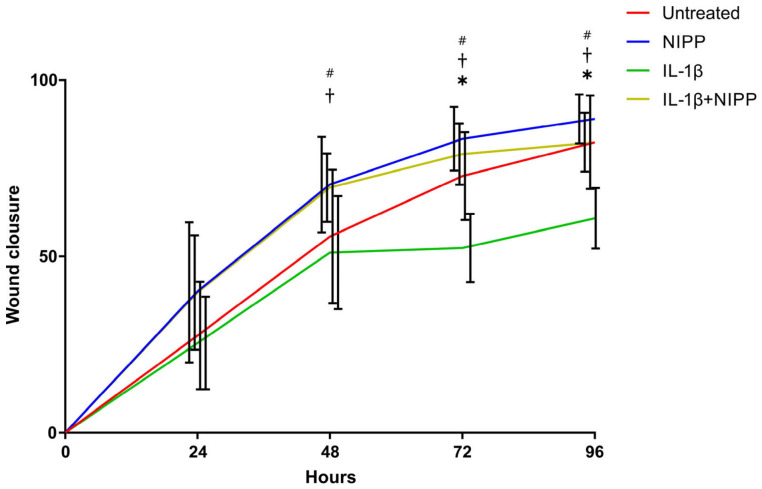
In vitro wound closure of hPDLC monolayers over 96 h, assessed by scratch assay. Untreated cells (red), NIPP-treated cells (blue), IL-1β–treated cells (green), and IL-1β + NIPP–treated cells (yellow). NIPP was applied for 120 s and IL-1β at 1 ng/mL. *n* = 15. * Significantly different between IL-1β and control; # significantly different between NIPP and IL-1β; † significantly different between IL-1β + NIPP and IL-1β (*p* < 0.05).

**Figure 5:**
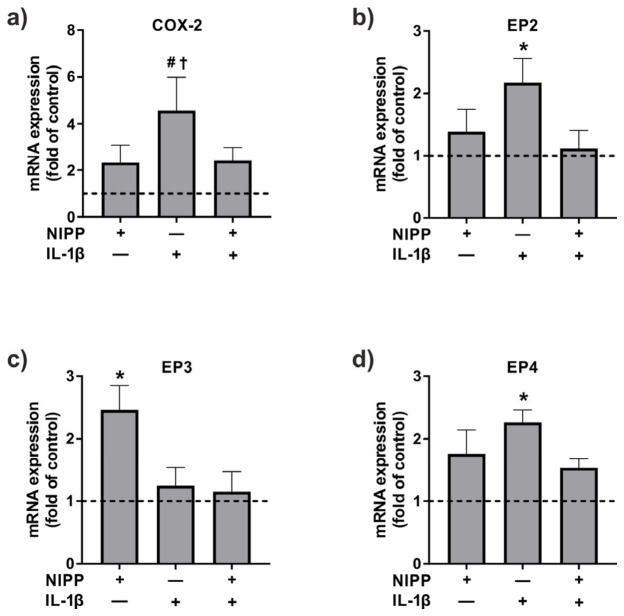
Effect of NIPP (120 s) on the gene expression of *COX-2* (**a**), *EP2* (**b**), *EP3* (**c**), and *EP4* (**d**) in hPDLC in the presence and absence of IL-1β (1 ng/mL) after 24 h. Untreated cells served as the control (dashed line). “+” indicates the presence and “−” the absence of the respective treatment. *n* = 15. * Significantly different from all other groups; # significantly different from control; † significantly different from the combined NIPP and IL-1β group (*p* < 0.05).

**Figure 6:**
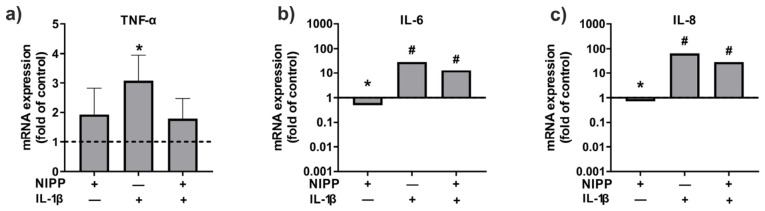
Effect of NIPP (120 s) on the gene expression of TNFα (**a**), *IL-6* (**b**), and *IL-8* (**c**) in hPDLC in the presence and absence of IL-1β (1 ng/mL) after 24 h. Untreated cells served as the control (dashed line). “+” indicates the presence and “−” the absence of the respective treatment. *n* = 15. * Significantly different from all other groups; # significantly different from control (*p* < 0.05).

**Figure 7:**
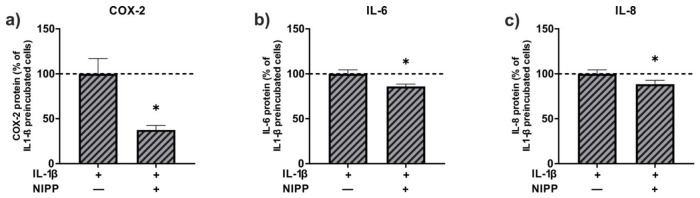
Effect of NIPP (120 s) on the protein levels of COX-2 (**a**), IL-6 (**b**), and IL-8 (**c**) in hPDLCs in the presence of IL-1β (1 ng/mL) after 24 h. Protein levels of IL-1β-incubated cells were normalised to 100% (indicated by the dashed line). “+” indicates the presence and “−” the absence of the respective treatment. *n* = 12. * Significantly different from IL-1β group (*p* < 0.05).

**Figure 8:**
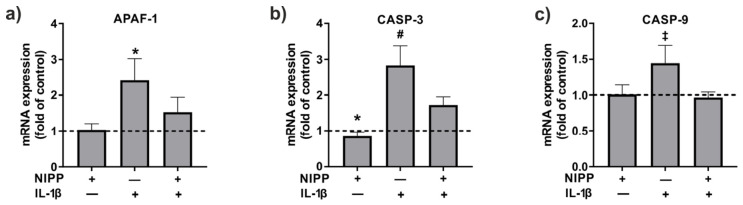
Effect of NIPP (120 s) on the gene expression of *APAF-1* (**a**), *CASP-3* (**b**), and *CASP-9* (**c**) in hPDLC in the presence and absence of IL-1β (1 ng/mL) after 24 h. Untreated cells served as the control (dashed line). “+” indicates the presence and “−” the absence of the respective treatment. *n* = 15. * Significantly different from all other groups; # significantly different from control; ‡ significantly different from the NIPP group (*p* < 0.05).

**Figure 9:**
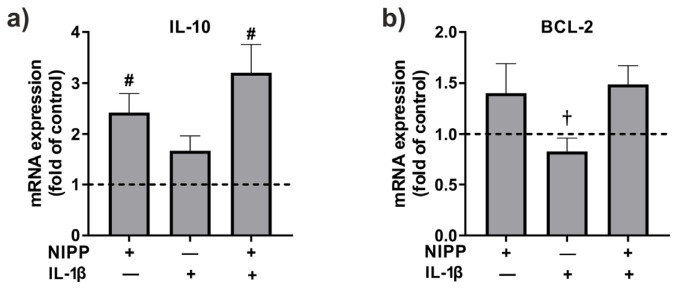
Effect of NIPP (120 s) on the gene expression of *IL-10* (**a**), and *BCL-2* (**b**) in the presence and absence of IL-1β (1 ng/mL) after 24 h. Untreated cells served as the control (dashed line). “+” indicates the presence and “−” the absence of the respective treatment. *n* = 15. † significantly different from the combined NIPP and IL-1β group, # significantly different from control (*p* < 0.05).

## Data Availability

The original contributions presented in this study are included in the article. Further inquiries can be directed to the corresponding authors.
